# Dental Inspection and Treatment of School Children

**Published:** 1908-03-15

**Authors:** R. M. Capon

**Affiliations:** Glasgow, Scotland


### DENTAL INSPECTION AND TREATMENT OF SCHOOL CHILDREN.

BY R. M. CAPON, L.D.S., GLASGOW, SCOTLAND.

That the condition of the teeth of school children is in a very bad way is generally recognized by those who have to do with child life. For expert confirmation we have but to examine the statistics in the "Memorandum" published by the British Dental Association where it is shown that in the mouths of 10,517 boys and girls of an average age of 12 years there were no less than 37,105 unsound teeth. If we take these statistics as a standard for comparison we should expect to find about 500,000 decayed teeth in the mouths of the children attending the Elementary Schools in our city. Unfortunately that section of the public who have to pay do not know these facts, nor what a serious loss it entails. For economical reasons, as I hope to show, dental inspection is likely to prove the wisest economy. If we can but demonstrate this, we are less likely to hear of objections to spending public monies on the teeth of our school children.

It is already said that too much is spent on education, and I am disposed to agree with Sir James Barr, who in a letter to me on this subject, said "If there were a little more money spent on the health and a little less on the education of the rising generation, it would be much better for the nation."

But the question is not so much what we spend, but what value we receive for the expenditure. Suppose, for example, the yearly cost to Liverpool be one pound per child. Do we get an equitable return. I maintain that we do not. As has been said by a keen observer "The loss of educational time from study either from actual absence from school, or from the overmastering tyranny of toothache, in the sturdy youngster who will not absent himself even for this fell fiend, the tear-stained face of the little maiden or the unsightly swollen cheeks are too common subjects for our consideration." The same authority further remarks:—"I do not hesitate to say that my experience warrants me in stating that 30 per cent of the ailments of boys and girls in good normal healthy circumstances are due to dental conditions that could be prevented; and in children of less robust nature the percentage is much higher."

This is the experience of a teacher, examiner and Educational Director of one of our large cities, an experience which we as dentists can confirm. Children are continually under our care who have become absolutely demoralized by the incessant pain and the weariness brought on by decayed teeth, and I can well imagine instances where the whole life is embittered by such a cause. I think of the boy who is keen in his work, keeps ahead in his form, and promises well, but who fails in the race, simply through the want of a little dental attendance. Under present conditions the teacher is unable to help the boy, parents may be unable to pay a dentist, and the pressure of home duties makes it often impossible to take him to the Dental Hospital, and so the poor child is allowed to drift on. Is it any wonder such a boy becomes a "Slacker?" The moral effect must be taken into account when considering the value of what is got for the money spent. Further, large sums are spent on sanitation, magnificent buildings with well-constructed school rooms are erected, at the same time the atmosphere in these rooms is contaminated by the foul expired air of large numbers of children whose mouths are generally in a most neglected condition, and we all know how difficult it is to exert our mental powers in an impure atmosphere.

But perhaps the most wasteful expenditure is that on Industrial and other schools, where the boys look to the Army or Navy as their future occupation. I have lately been inspecting the teeth of boys, over 200 in number, in an Industrial School in Liverpool. A more promising dentally equipped set of boys it would be impossible to find anywhere. I have brought the charts of a number of them for your inspectoin. Granted that in most cases their mouths are very dirty, many of their teeth are decayed, but the ravages of decay have not gone so far, but what at a very small cost the teeth could be put into very excellent condition, and would remain in this state because most of the boys have passed the age when teeth are so liable to decay. Many of these boys will present themselves at the proper age as recruits for medical examination. The expenditure of a few pounds now would make it possible for every boy in this school, other things permitting, to pass that examination, but if neglected, I can safely say not 10 per cent would pass.

The consequence is, we have large numbers of rejections of able-bodied men. It is neither more or less than a grave public scandal that such large sums are spent on training boys for these professions, and after all being obliged to refuse their services. The army reports show that rejections due solely to the condition of the teeth had risen during the years 1891 to 1903 from 10.88 per cent to 49.26 per cent and we are told by Sir Wm. Taylor, medical officer to the army, that the medical expenditure during the late war was increased enormously by the fact of having to supply artificial teeth to our troops. It is further said that the return of 3,000 recruits from the seat of war cost the Government £25,000, a return only accounted for by the fact that the teeth of those soldiers were so bad that they were unable to eat the rations supplied to the regular troops. It is therefore proved that there are economical reasons for Dental Inspection and treatment. This so impressed itself on the Inter-Departmental Committee on Physical Deterioration, the War Office Inter-Departmental Conference, and the British Dental Association.

That the following recommendations were submitted to the Board of Education:—

1. "That the teaching of the elements of hygiene should be made compulsory in schools, and in this teaching the care of the teeth should receive special attention."
2. "That daily cleansing of the teeth should be enforced by parents and teachers."
3. "That systematic examination of the teeth of children by competent dentists, employed by school authorities, should be practised where possible. To prevent caries extending, to stop carious teeth, and to remedy defects of the teeth."

The effects of these recommendations, and the pressure of public opinion through Congresses on Hygiene, Health Conferences, etc., was followed by the passing of the Act of last Session and the consequent establishment of the Medical Department before mentioned.

The immensity of the task which this Department has before it can be measured, if it is remembered that in Liverpool alone there are 132,325 children on the rolls, all of whom will have to be inspected. A grave public duty, therefore, devolves upon this and kindred societies. There will be no getting away from the fact that we shall be called upon to help the Education Committee to formulate a scheme whereby dental inspection and possibly treatment can be efficiently carried out.

In anticipation of this being the case here in Liverpool, I have thought it proper to submit the following, in the hope that a free discussion will enable this Society to recommend one that will meet with the requirements of the Education Committee. It would be well to bear in mind that this is a new departure, therefore proposals should be moderate both in scope and cost.

On looking over the foregoing recommendations, as submitted to the Board of Education, you will notice that daily cleansing of the teeth is advised, and is the keynote of all of them. The reason for this is because all evidence clearly showed that the extraordinary prevalence of caries was not due to any physical deterioration, but to want of attention to the mouth, especially during the period of childhood. Therefore our first duty should be to attempt to inculcate habits of cleanliness particularly in regard to the regular brushing of the teeth.

This should be done by "simple plain instruction in language children can understand." This instruction cannot, in my opinion, be better imparted than by the chart which you have before you, which simply, plainly and clearly informs the child how to keep his teeth. How to avoid having bad teeth. How to clean and when to clean his teeth. The chart will also be useful as a guide to teachers who, without special knowledge, can enlarge upon the several points during the regular lecture course of school hygiene.

### GOOD TEETH MEAN GOOD HEALTH.

### How To keep Your Teeth.

Teeth decay because food is not cleaned away from them after meals.

Decay begins on the outside and does not pain you. Next it gets to the nerve and you have toothache, and perhaps an abscess and a swollen face.

If your teeth are bad, you cannot chew properly your food does you little good. This is bad; but it is not the worst. The worst is that bad teeth poison the whole body; frequently they cause dangeruos illnesses.

If your teeth are decayed, they should have attention. The sooner the better. *It is foolish to wait till they ache.*

### HOW TO AVOID HAVING BAD TEETH.

You must keep your teeth clean.

### HOW TO CLEAN YOUR TEETH.

With a small tooth brush and common whiting (prepared chalk), you can get from the chemist enough whiting for a penny to last months.

The reason why the brush must be small is because you want to get into all the corners; you must brush your teeth on all their sides, and you must, above all, remember to work the bristles of the brush upwards and downwards in between the teeth, because it is there that the food sticks and does all the harm.

Rinse your mouth out with water when the brushing is finished.

### WHEN TO CLEAN YOUR TEETH.

The best time is after your last meal. If you go to bed with a dirty mouth your teeth are in danger all night. If you can manage to brush your teeth after every meal so much the better for you.

Chewing hard food is a great help to the teeth.

If you take care of your teeth it will help you to grow up strong and healthy.

In order to speedily introduce these charts into schools, I have arranged with Messrs. Philip, Son & Nephew, Ltd., to send a copy, together with an introductory prospectus, to every Educational Committee throughout England and Wales; so far it has been highly approved by eminent authorities and the National Union of Women Workers, Liverpool, branch unanimously passed the following resolution in regard to it:—

"That this meeting of the Liverpool branch of the N. U. W. W. approves of the chart, "Good teeth mean good health," and recommends it to the consideration of elementary education authorities with a view to its being hung up in every classroom in public elementary schools."—*Dental Record.*

